# Sperm Imprinted Gene Methylation and DNA Fragmentation in ICSI Outcomes: A Pilot Study

**DOI:** 10.3390/epigenomes10020032

**Published:** 2026-05-10

**Authors:** Anna Chiara Conflitti, Fani Konstantinidou, Alessandra Buonacquisto, Gaia Cicolani, Enrico Delli Paoli, Silvia Di Chiano, Antonella Linari, Ludovico Muzii, Serena Bianchini, Federica Quaranta, Francesco Pallotti, Francesco Lombardo, Liborio Stuppia, Valentina Gatta, Donatella Paoli

**Affiliations:** 1 Laboratory of Seminology and Sperm Bank “L. Gandini”, Department of Experimental Medicine, “Sapienza” University of Rome, 00161 Rome, Italy; annachiara.conflitti@uniroma1.it (A.C.C.); alessandra.buonacquisto@uniroma1.it (A.B.); gaia.cicolani@uniroma1.it (G.C.); enrico.dellipaoli@uniroma1.it (E.D.P.); silvia.dichiano@uniroma1.it (S.D.C.); serena.bianchini@uniroma1.it (S.B.); federica.quaranta@uniroma1.it (F.Q.); francesco.lombardo@uniroma1.it (F.L.); donatella.paoli@uniroma1.it (D.P.); 2 Department of Neuroscience, Imaging and Clinical Sciences, School of Medicine and Health Sciences, “G. d’Annunzio” University of Chieti-Pescara, 66100 Chieti, Italy; stuppia@unich.it (L.S.); v.gatta@unich.it (V.G.); 3 Unit of Molecular Genetics, Center for Advanced Studies and Technology (CAST), “G. d’Annunzio” University of Chieti-Pescara, 66100 Chieti, Italy; 4 Department of Maternal and Child Health and of Gynecological Sciences, “Sapienza” University of Rome, 00161 Rome, Italy; antonella.linari@uniroma1.it (A.L.); ludovico.muzii@uniroma1.it (L.M.); 5 Department of Medicine and Surgery, University of Enna “Kore”, 94100 Enna, Italy; francesco.pallotti@unikore.it; 6 Diabetology and Endocrinology Unit, Hospital “Umberto I”, Azienda Sanitaria Provinciale di Enna, 94100 Enna, Italy

**Keywords:** sperm DNA methylation, H19, IGF2, PEG1/MEST, sperm DNA fragmentation, embryo development

## Abstract

Background/Objectives: Aberrant DNA methylation of imprinted genes and increased sperm DNA fragmentation (SDF) have been implicated in male infertility. However, their impact on assisted reproductive technology (ART) outcomes remains unclear. This pilot study aimed to investigate SDF and methylation status of H19, IGF2, and PEG1/MEST in relation to fertilisation and embryo development following intracytoplasmic sperm injection (ICSI). Methods: Twenty male partners of women undergoing ICSI were recruited and classified according to ART outcome into viable embryos (VEs, n = 7), non-viable embryos (NVEs, n = 7), and no fertilisation (NF, n = 6). Before sperm selection, an aliquot of each seminal sample was used for semen analysis according to WHO, 2021, SDF assessment (TUNEL assay), and sperm DNA methylation analysis of H19, IGF2, and PEG1/MEST (pyrosequencing). Results: Semen parameters were above the fifth percentile. SDF was significantly lower in the VE group compared with the other groups. H19 CpG1 methylation correlated positively with viable embryos (*p* = 0.016), while H19 CpG2 island showed a positive correlation with sperm concentration (*p* = 0.028). In male/couple infertility cases, total H19 methylation correlated negatively with SDF (*p* = 0.050). IGF2 CpG3 island methylation correlated positively with viable embryos (*p* = 0.027). Total PEG1/MEST methylation was positively correlated with fertilisation events (*p* = 0.002) and viable embryos (*p* = 0.011). PEG1/MEST CpG2 island also positively correlated with sperm motility (*p* = 0.034), while CpG3 and CpG4 showed significant correlations with fertilisation (*p* < 0.001; *p* = 0.004). Conclusions: This pilot study shows that SDF and sperm methylation levels of H19, IGF2, and PEG1/MEST are related to ICSI outcomes, supporting that sperm molecular and epigenetic features may influence fertilisation and embryo development.

## 1. Introduction

Epigenetic modifications play a crucial role in the genomic reprogramming process that occurs during early embryo development. After fertilisation, parental alleles undergo extensive demethylation, with the exception of imprinted genes, which retain their patterns [1]. De novo DNA methylation begins at the blastocyst stage and its maintenance during the post-implantation phase is essential for proper embryonic development [2]. This dynamic process represents the epigenetic reprogramming phase, during which each cell develops a specific methylation profile. DNA methylation reprogramming occurs in two main waves: the first involves the gametes, and the second occurs during pre-implantation development. Imprinted genes escape the second wave of reprogramming, preserving their parent-of-origin-specific expression [1]. These marks in germline genes play a key role in ensuring correct spermatogenesis. Among imprinted genes, H19, IGF2 (insulin-like growth factor 2), and PEG1/MEST (paternally expressed gene 1/mesoderm-specific transcript) are particularly relevant for human male reproduction. The H19 and IGF2 genes are located on human chromosome 11p15.5 and share a common imprinting control region (ICR). H19 plays a role in early growth and differentiation, while IGF2 codes for insulin-like growth factor 2 involved in cellular proliferation, foetal and placental development, and organogenesis [3,4]. Under normal imprinting conditions, on the maternal allele, the Differentially Methylated Region (DMR) is hypomethylated, allowing CTCF (CCCTC-binding factor) to bind the ICR. This prevents enhancer access to IGF2, thereby silencing its expression and permitting H19 expression [1]. Conversely, the paternal allele is hypermethylated at both DMR and ICR, permitting IGF2 activation and silencing H19 [5]. Thus, H19 is maternally expressed, whereas IGF2 gene is paternally expressed. PEG1/MEST, located on chromosome 7q32, is paternally expressed and typically unmethylated in sperm, whereas the maternal allele is methylated and transcriptionally inactive. This gene encodes a member of the α/β-hydrolase protein family, which is involved in early development [6], mesodermal development and cellular growth [7]. Alterations in the imprinting of PEG1/MEST have been associated with various types of cancer and other pathological conditions [6,8].

Aberrant methylation of imprinted genes has been reported in spermatozoa of infertile men, particularly in conditions such as oligospermia [9,10,11,12] and azoospermia [13], where hypomethylation of the H19 gene and hypermethylation of PEG1/MEST gene have been observed. These findings support a role for epigenetic dysregulation in impaired spermatogenesis and sperm function. Moreover, as epigenetic alterations in germ cells may be transmitted to the offspring, imprinting defects could also affect embryo development. Despite these observations, the clinical impact of sperm DNA methylation on assisted reproductive technology (ART) outcomes remains unclear [1]. Available evidence is limited and often inconsistent, with conflicting results across studies. Most studies have focused on global methylation or overall gene methylation levels, without analysing individual CpG sites within gene promoters. In addition, their relationship with clinically relevant outcomes, including fertilisation rate, embryo development, and sperm DNA fragmentation (SDF), remains poorly explored. In particular, data in the context of intracytoplasmic sperm injection (ICSI) are still scarce and heterogeneous.

The aim of our study was therefore to evaluate SDF and the methylation status of specific CpG sites within the H19, IGF2, and PEG1/MEST genes in spermatozoa in relation to fertilisation and embryo development following ICSI.

## 2. Results

### 2.1. Patients

Twenty male partners of women undergoing ICSI cycles were recruited. The selected subjects were classified into three categories based on the outcome of ART: 6 did not achieve fertilisation (NF), 7 achieved fertilisation but the embryo was not viable (NVE), and 7 obtained a viable embryo (VE). The main causes of infertility were the presence of a female factor (either ovulatory or tubal factor) in 12/20 couple (60%), a male factor in 5/20 couple (25%), and a combined factor (couple infertility) in 3/20 (15%). Table 1 presents demographic characteristics of both male and female partners of recruited couples. Male age and Body Mass Index (BMI) of the NF group appeared to be significantly higher than the other groups (Table 1).

### 2.2. Semen Parameters and Sperm DNA Fragmentation (SDF)

**Figure 1:**
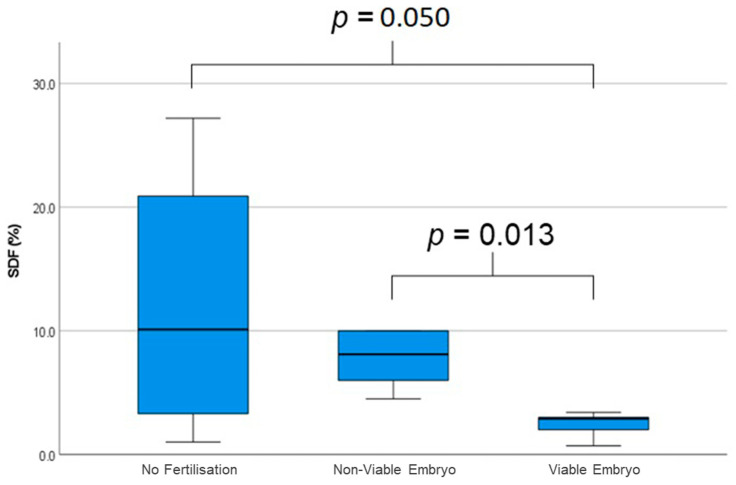
Percentage of sperm DNA fragmentation (SDF) in three groups.

Semen characteristics and SDF (%) are reported in Table 2. No significant differences were observed among the groups in terms of sperm concentration, percentage of progressive motility, abnormal morphology, or sperm vitality. However, a significant reduction in SDF (%) was found in the VE group compared to both NVE (*p* = 0.013) and NF (*p* = 0.050) groups. Higher SDF levels were correlated with fertilisation failure or embryo development arrest, while lower levels were observed in cases leading to viable embryos (Figure 1).

Spearman’s correlation analysis revealed that SDF (%) was negatively correlated with sperm concentration (*Rho* = −0.682, *p* = 0.003), progressive motility (*Rho* = −0.641, *p* = 0.007), and sperm vitality (*Rho* = −0.730, *p* = 0.001). In contrast, a positive correlation was found between SDF (%) and abnormal forms (%) (*Rho* = 0.689, *p* = 0.002). After adjustment for age and BMI, we detected a negative correlation between SDF (%) and fertilisation rate (*Rho* = −0.622, *p* = 0.013), and a positive association between progressive motility and fertilisation rate (*Rho* = 0.499, *p* = 0.035). (Table 3).

### 2.3. Methylation Analysis of H19 Gene Promoter

The methylation profile of the four CpG islands within the H19 gene promoter in sperm DNA was analysed. Table 4 shows the results of the epigenetic analysis across the three groups (Table 4). Higher total H19 methylation levels were observed in the viable embryo (VE) group compared to the others, although this difference did not reach statistical significance. When considering subgroups based on male infertility and couple-related factors, statistical analysis revealed a significant negative correlation between total H19 methylation and SDF (%) (*Rho* = −0.949, *p* = 0.05).

Among the four CpG islands, CpG4 was found to be fully methylated (100%) in all groups. In particular, CpG1 and CpG2 islands showed interesting results:

- CpG1 island—The methylation in this island showed lower values in the NF group, with a gradual increase through NVE and the highest levels in VE, although the differences were not statistically significant (Table 4). Spearman’s correlation analysis revealed a significant positive association between CpG1 methylation and the presence of a viable embryo, which remained significant after adjustment (Table 5).
- CpG2 island—Differences in methylation among the three groups were not statistically significant. However, a significant positive correlation was found between CpG2 methylation and sperm concentration (*Rho* = 0.503, *p* = 0.028).

### 2.4. Methylation Analysis of IGF2 Gene Promoter

The methylation profile of the three CpG islands located near the IGF2 gene promoter in sperm DNA was analysed. Table 6 shows the methylation levels for each CpG island across the three groups (Table 6). Overall, methylation percentages were similar between the NF and NVE groups, while a slight increase was observed in the VE group. However, this difference did not reach statistical significance. Total IGF2 methylation showed a positive correlation trend with sperm vitality (*Rho* = 0.404, *p* = 0.078) and a negative trend with SDF (%) (Table 7). When analysing individual CpG islands, Spearman’s correlation revealed several results that, while not all statistically significant, may be biologically relevant:

- CpG1 island—A negative correlation trend was observed between SDF (%) and CpG1 methylation (Table 7);
- CpG2 island—Positive correlation trends were found between CpG2 methylation and sperm concentration (*Rho* = 0.428, *p* = 0.060) as well as fertilisation (Table 7);
- CpG3 island—The methylation percentage of CpG3 was significantly positively correlated with viable embryos (Table 7). Additionally, a positive trend between CpG3 and fertilisation rate was observed (Table 7).

### 2.5. Methylation Analysis of PEG1/MEST Gene Promoter

The methylation profile of four CpG islands located near the promoter of the PEG1/MEST gene in sperm DNA was analysed. Table 8 shows the methylation percentages for each CpG island in the three groups (Table 8). Total methylation differed significantly across three categories (*p* = 0.007). Specifically, total methylation (%) was lowest in the NF group, intermediate in NVE, and highest in VE. By dividing the groups into two categories—fertilisation (viable and non-viable embryos) and no fertilisation—a significant increase in the methylation percentage was observed in the fertilisation group (NVE and VE) compared to the no fertilisation group (*p* = 0.003). This difference was confirmed by directly comparing VE and NVE (*p* = 0.014). Statistical analysis also revealed that total PEG1/MEST methylation was significantly and positively correlated with both fertilisation and viable embryos (Table 9). Regarding individual CpG islands:

- CpG2 island—A significant positive correlation was identified between CpG2 methylation and both progressive sperm motility (Rho = 0.475, *p* = 0.034) and viable embryos (Table 9). A negative correlation trend was also observed between CpG2 methylation and SDF (%) (Table 9).
- CpG3 islands—CpG3 methylation showed a significant positive correlation with viable embryos and a strong correlation with fertilisation (Table 9). In addition, a significant and positive correlation was observed between CpG3 methylation and sperm vitality percentage (*Rho* = 0.463, *p* = 0.040);
- CpG4 island—A significant positive correlation was observed between CpG4 methylation percentage and fertilisation rates (Table 9). CpG4 methylation was also positively correlated with sperm vitality percentage, although this did not reach statistical significance (*Rho* = 0.435, *p* = 0.055).

## 3. Discussion

Despite significant advances in diagnostic methodologies, the underlying cause of male infertility often remains elusive. Previous research has focused on genetic defects, but these alterations explain only 15–30% of male infertility cases [14,15]. In recent years, increasing attention has been given to sperm DNA damage and epigenetic dysregulation as potential contributors to male reproductive failure [16]. Since epigenetic mechanisms influence gene expression without altering the DNA sequence [2], they could explain at least some cases of male infertility with no apparent cause. In this context, epigenetic studies provide new insights into infertility, contributing to our understanding of its causes and management [2]. Among these mechanisms, DNA methylation plays a crucial role in genomic imprinting. While previous research has suggested a potential involvement of DNA methylation in male infertility, its contribution to ART outcomes is still being debated. For this reason, the present study investigated the impact of sperm DNA methylation on ICSI outcomes. In particular, we focused on the H19, IGF2, and PEG1/MEST genes, which have previously been shown to exhibit altered methylation patterns in various forms of spermatogenic impairment. We also analysed the effect of SDF on ICSI outcomes, given that DNA damage may affect embryo development. Our findings support the hypothesis that sperm epigenetic alterations, together with DNA fragmentation, may influence fertilisation success and early embryo development following ICSI. This is consistent with the emerging concept that spermatozoa contribute to embryo competence beyond the delivery of paternal DNA through epigenetic and molecular signals. Interestingly, we observed that increasing male age was associated with poorer reproductive outcomes, with age being significantly higher in the NF group compared with the VE group. This observation is consistent with the findings of other studies that suggest an increase in paternal age affects the outcomes of ART [17,18,19]. The present study provides further insight into the role of sperm epigenetic and molecular features in ART.

### 3.1. Semen Parameters and Sperm DNA Fragmentation (SDF)

In our cohort, semen parameters were above the fifth percentile according to the WHO (2021) [20]. After adjustment for potential confounders, progressive motility emerged as significantly and positively correlated with fertilisation rate.

In recent years, growing attention has focused on molecular markers of sperm quality. Sperm DNA fragmentation (SDF), particularly when assessed by the TUNEL assay, has been suggested as a reliable indicator of good semen quality [21,22]. In our study, higher SDF levels were strongly correlated with reduced sperm concentration, progressive motility, sperm vitality, and increased abnormal morphology. Moreover, after adjustment for confounders, SDF showed a significant negative correlation with fertilisation rate, further supporting its role in sperm functional competence. We emphasise that the direction and magnitude of the associations observed after adjustment are consistent with established biological mechanisms. In particular, the negative correlation between SDF and fertilisation rate, together with the positive association between progressive motility and fertilisation, reflects the interplay between sperm DNA integrity and functional sperm quality. The strengthening of these associations after adjustment may reflect a reduction in confounding variability, allowing underlying relationships to emerge more clearly. Given the exploratory nature of the study and the small sample size, these findings should be interpreted with caution, but they are consistent with the existing literature. Sun et al. (1997), in a study involving 298 infertile men, demonstrated that samples with reduced semen parameters and poor fertilisation outcomes had higher SDF levels [23]. Moreover, we observed that elevated SDF was significantly correlated with embryo development arrest, whereas lower SDF levels were characteristic of samples that resulted in viable embryos. A systematic review reported that sperm DNA damage adversely affects fertilisation rates, embryo quality, blastocyst formation, implantation, pregnancy rates, and live birth rates in in vitro fertilisation (IVF) treatments [24]. The impact of SDF on reproductive outcomes is further supported by its association with recurrent pregnancy loss [25]. These observations are also in agreement with our previous study [26], in which post-sperm selection SDF was positively correlated with the percentage of low-quality embryos and negatively correlated with the formation of viable embryos. Of particular relevance, SDF > 2.9% increased the risk of obtaining a non-viable embryo by almost four-fold. While some controversy remains regarding the predictive value of SDF in ICSI, our data reinforce the concept that sperm DNA integrity plays a critical role in embryo developmental competence in ART.

### 3.2. Methylation of H19, IGF2, PEG1/MEST Genes in Relation to ICSI Outcomes

Differential methylation of CpG islands in imprinted genes constitutes an epigenetic signature that distinguishes paternal from maternal allele. The methylation patterns of H19 and IGF2 are established during the adult spermatogonial phase following erasure of their imprint during foetal development. In particular, the H19 gene is transcriptionally active on the maternal allele, which remains unmethylated, and silenced on the methylated paternal allele. Its methylation status is closely linked to the expression of the adjacent IGF2 gene, which is paternally expressed and regulated by the same imprinting control region. Conversely, PEG1/MEST is expressed from the paternal allele, which remains largely unmethylated during spermatogenesis. Its promoter undergoes demethylation during germ cell epigenetic reprogramming and remains unmethylated in mature spermatozoa. As expected, we found that H19 and IGF2 exhibited higher methylation percentages compared with PEG1/MEST in line with their imprinting status. Notably, the CpG sites analysed in this study are located within well-characterised regulatory regions of imprinted genes, including promoter-associated regions and DMRs, which are essential for the establishment and maintenance of imprinting marks. These regions play a key role in controlling allele-specific gene expression and are known to be susceptible to epigenetic alterations in different biological and pathological contexts.

H19 gene—Our study provides further evidence that altered methylation of the paternal imprinted H19 gene in sperm DNA may influence fertility and the success of ART outcomes. We observed a positive correlation between H19 CpG1 methylation and viable embryos, suggesting that appropriate methylation at this site is essential for embryonic development. This finding aligns with previous studies reporting that proper methylation of H19 is crucial for embryonic development, and its alteration can cause miscarriage [27]. These data suggest that defects in the methylation of the imprinted H19 gene could negatively affect embryo development. In addition, we identified a significant positive association between methylation at H19 CpG2 and sperm concentration, consistent with previous studies linking H19 hypomethylation to oligozoospermia [12,28,29] and azoospermia [30]. Finally, we also found a significant inverse correlation between overall H19 methylation and SDF (%) in cases of male or combined infertility. This supports the hypothesis that altered imprinting contributes to genomic instability. Another study has shown that there is a negative relationship between global sperm DNA methylation levels and both sperm chromatin condensation and DNA integrity [31]. However, few studies have assessed the relationship between H19 gene methylation and sperm DNA integrity, and the results are inconsistent. Ni et al. (2019), using the Sperm Chromatin Structure Assay (SCSA), found no significant association between H19 gene methylation and sperm DNA damage [32]. Another study did not observe a direct association between H19 gene methylation and SDF assessed by the sperm chromatin dispersion (SCD) test. The inconsistent results reported in the literature may be due to the use of different assays. In our study, we used the TUNEL assay, a method that directly detects DNA strand breaks.

Our findings highlight a possible involvement of H19 methylation in fertility processes, including sperm production and ICSI outcomes. Supporting this interpretation, a recent systematic review [33] has shown that altered H19 methylation is associated with poorer ART outcomes and a higher risk of pregnancy loss. Given that H19 expression is inversely related to cell growth and inhibits cell proliferation [34], alterations in its methylation could impair spermatogenesis and reduce embryo developmental competence.

IGF2 gene—The IGF2 gene is a paternally imprinted gene whose expression is tightly regulated by the methylation status of H19 locus. Yamaguchi et al. (2019) highlighted that hypomethylation of the H19 DMR can alter the H19/IGF2 expression ratio at the post-zygotic level, contributing to the pathogenesis of pregnancy complications [35]. For this reason, our study also aimed to investigate the potential relationship between IGF2 methylation and reproductive outcomes in ICSI. We analysed the methylation profile of the three CpG islands located near the IGF2 promoter. In our study, total IGF2 methylation was slightly higher in the viable embryo group compared with the other groups, although this difference did not reach statistical significance. Importantly, when individual CpG islands were considered, CpG3 methylation was significantly and positively associated with the number of viable embryos. Our findings support a role for IGF2 methylation in early embryonic development, in agreement with evidence showing that IGF2 is exclusively paternally expressed and spermatozoa represent the only source of this growth factor to the oocyte, thereby potentially regulating this process [36]. Additionally, CpG2 methylation showed non-significant positive trends with sperm concentration. Our data should be interpreted with caution, despite this finding being consistent with previous evidence indicating that IGF family growth factors produced by Sertoli cells are involved in spermatogonial development and can influence sperm production [36]. Thus, our data may support the hypothesis that adequate methylation of IGF2 may be important for maintaining normal spermatogenesis.

Furthermore, an inverse, non-significant trend was observed between IGF2 methylation and SDF, particularly at CpG1. This observation is in line with previous reports linking altered IGF2 methylation to increased SDF in infertile men [32]. Overall, our results suggest that certain CpG sites within the IGF2 promoter may be linked to spermatogenesis, embryonic development, and sperm chromatin integrity. However, given the lack of statistical significance, these findings should be considered exploratory and require confirmation in larger studies.

PEG1/MEST—PEG1/MEST is paternally expressed and generally considered unmethylated in mature sperm. In our cohort, low levels of methylation (mean of 2%, range 0–5%) were observed and were compatible with successful fertilisation and embryo development. Total methylation of the PEG1/MEST gene promoter was significantly higher in the group with viable embryos compared to both no fertilisation and non-viable embryo groups. Statistical analyses confirmed that total PEG1/MEST methylation positively correlates with fertilisation success and viable embryos, suggesting the potential relevance of this epigenetic mark in reproductive competence. This finding suggests that low levels of methylation may be physiologically tolerated, refining the conventional view of a fully unmethylated paternal allele. In addition, CpG2 methylation showed a significant positive correlation with progressive sperm motility. In contrast with previous studies reporting that PEG1/MEST hypermethylation reduced sperm motility [12], our results suggest that minimal methylation at specific CpG sites may support normal sperm function. CpG2 methylation showed an inverse, non-significant correlation with sperm DNA fragmentation. In parallel, CpG3 methylation was significantly associated with sperm vitality. While these findings do not demonstrate a direct relationship, they may indicate a possible role of PEG1/MEST methylation in the regulation of sperm chromatin integrity, which should be confirmed in future studies.

To date, no threshold value has been defined to distinguish normal and abnormal methylation [37,38]. A previous study [39] reported aberrant methylation patterns of PEG1/MEST in oligozoospermic men and proposed a reference range of 0–15% for normal methylation based on normozoospermic controls. In our study, the observed methylation levels were lower than those reported by Kläver et al. (2013) [39], despite all patients being normozoospermic. Our data show an average total methylation of around 2%, with values ranging from 0 to 5%. Overall, our data suggest that complete demethylation of the paternal allele may not be required, and that low levels of methylation may be compatible with normal spermatogenesis and embryo development. Together with existing evidence, these data may help define methylation ranges for the PEG1/MEST gene. However, further validation in larger studies is required.

## 4. Materials and Methods

### 4.1. Patients

Men whose female partners underwent an ICSI cycle were recruited. The inclusion criteria for the couples were as follows: male partners aged between 18 and 50 years; female partners aged between 18 and 43 years; the presence of mature oocytes suitable for insemination; and the possibility to provide a sufficient aliquot of semen sample for molecular analyses. In contrast, couples with the following conditions were excluded from the study: endometriosis for female partners; history of recurrent pregnancy loss; male partners with andrological conditions (e.g., cryptorchidism, clinically significant varicocele, hypogonadism) and/or endocrinological conditions that could affect sperm quality; azoospermia (both obstructive and non-obstructive); genetic syndromes and/or abnormal karyotypes; previous treatment with chemotherapy/radiotherapy and/or potentially gonadotoxic drugs for oncological conditions; and previous treatment with potentially gonadotoxic drugs for non-oncological conditions. Additionally, in the three months preceding the study, all recruited subjects had not undergone medical or surgical treatment and had no conditions (e.g., fever) that could interfere with semen analysis.

On the day of oocyte retrieval, all recruited subjects collected a semen sample. An aliquot of semen, in excess of that required for the ICSI procedure and collected prior to sperm selection, was used for the following analyses: semen analysis, SDF, and evaluation of methylation levels of the H19, IGF2, and PEG1/MEST genes.

### 4.2. Assisted Reproductive Procedures

Ovarian stimulation followed a GnRH antagonist protocol (Fyremadel 0.25 mg, Ferring, Saint-Prex, Switzerland), starting on day 2 of the treatment cycle with administration of 150 to 225 urinary FSH + LH (Meropur, Ferring, Saint-Prex, Switzerland), according to ovarian reserve test results. From day 6 of stimulation, follicular development was monitored daily by ultrasound, and plasma concentrations of oestradiol and progesterone were measured. The criteria used for triggering ovulation with 10,000 IU human chorionic gonadotropin (Gonasi HP 5000, IBSA, Lugano, Switzerland) s.c. included: at least two follicles with a mean diameter > 17 mm (two perpendicular measurements), plasma E2 between 1000 and 3000 pg/mL, and plasma progesterone < 1.5 ng/mL. Oocyte retrieval was performed 36 h after hCG administration by transvaginal US-guided follicular aspiration under intravenous sedation. Sample selection for our study was performed according to the Italian law governing ART at the time of the pick-up (law 40/2004 and subsequent amendments). At the time of follicular aspiration, metaphase II (MII) oocytes surrounded by expanded cumulus–corona cells were identified by phase-contrast microscopy and subsequently inseminated by ICSI. Fertilisation was assessed the following day, and on day 3 post-insemination, embryos were scored based on cell morphology, degree of fragmentation, and presence of multinucleation. Embryo transfer, guided by ultrasound, was performed between 72 and 120 h after insemination. The luteal phase was supported with the daily vaginal administration of 600 mg of progesterone.

According to the criteria established by the Istanbul Consensus (2011) [40], embryos were scored and classified in three grades. Grade 1 embryos exhibited stage-specific cell size, <10% fragmentation, and no multinucleation. Grade 2 embryos displayed stage-specific cell size in the majority of cells, 10–25% fragmentation, and no multinucleation. Grade 3 embryos were defined by non-stage-specific cell size, >35% of fragmentation, and evidence of multinucleation. In the present study, embryo quality was grouped into two categories as viable embryos and non-viable embryos—embryos in which development had been arrested for at least 24 h, or in which all cells had undergone degeneration or lysis [40]. The group of viable embryos includes those classified as Grades 1, 2, or 3 (also referred to as top, good, or low quality). These embryos were considered viable because they had the potential to develop and may be suitable for transfer.

### 4.3. Semen Analysis

Semen samples were collected by masturbation after 2–7 days abstinence. All samples were allowed to liquefy at 37 °C for 60 min and were then assessed according to the WHO (2021) [20]. The following variables were assessed: volume (mL), total sperm number (n × 106 per ejaculate), progressive motility (%), and morphology (% abnormal forms). Vitality tests were performed by eosin Y staining.

### 4.4. Sperm DNA Fragmentation (SDF)

SDF was evaluated using TUNEL (Terminal Deoxynucleotidyl Transferase dUTP Nick End Labelling) assay with in situ Cell Death Detection Kit, Fluorescein (Roche, Basel, Germany). An aliquot of each sample was centrifuged and processed as previously described by Paoli et al. 2019 [41]. The samples were then analysed under a fluorescence microscope counting at least 500 cells.

### 4.5. DNA Extraction from Spermatozoa

Before DNA extraction, semen samples were diluted in PBS to minimise the presence of non-spermatogenic elements. Sperm DNA extraction was performed using the Nucleospin Tissue—isolation of genomic DNA from semen (Macherey-Nagel, Düren, Germany). This method allows the selective separation of spermatozoa from contaminating cells through the use of Buffer GuEX (prepared according to the manufacturer’s instructions) and Proteinase K. In the first step, Buffer GuEX and Proteinase K were added to each sample, allowing lysis of round cells, epithelial cells, and leukocytes. After incubation and centrifugation, spermatozoa remained intact in the pellet, whereas DNA released from lysed contaminating cells was contained in the supernatant. Because this study focused specifically on sperm-derived DNA, only the pellets were processed for DNA extraction according to the manufacturer’s protocol.

### 4.6. Pyrosequencing

Genomic DNA from all samples was subjected to sodium bisulfite treatment as previously described [42], which converts unmethylated cytosines to uracils, and was stored at −20 °C until use. PCR amplification of the H19, IGF2, and PEG1/MEST promoters was performed using the 2× PyroMark PCR Master Mix and 10× CoralLoad Concentrate (Qiagen, Hilden, Germany), 0.2 µM of each primer, and 1 µL of bisulfite converted-DNA and nuclease-free water to a final volume of 25 µL. The conditions in the PCR stage were as follows: 95 °C for 15 min; 45 cycles at 94 °C for 30 s; an annealing temperature of 51 °C for 30 s (for H19 and IGF2) and 60 °C for 30 s (for PEG1/MEST); 72 °C for 30 s; and a final extension at 72 °C for 10 min. DNA methylation levels of all of the studied CpG sites (4 CpGs for H19, 3 CpGs for IGF2, 4 CpGs for PEG1/MEST) were assessed by pyrosequencing (Pyromark Q96ID, Qiagen, Hilden, Germany) and the PyroMark CpG software v.1.0.11 (Qiagen, Hilden, Germany). The PCR and pyrosequencing primers, as well as the analysed sequence, used were as follows:

- H19 Forward PCR Primer: 5′-TTTGTTGATTTTATTAAGGGAG-3′.

H19 Reverse PCR Primer: 5′-[Biotin]-CTATAAATAAACCCCAACCAAAC-3′.
H19 Sequencing Primer: 5′-GTGTGGAATTAGAAGT-3′.
Sequence to be analysed for H19:
GGTYGYGYGGYGGTAGTGTAGGTTTATATATTATAGTT.

- IGF2 Forward PCR Primer: 5′-TTTTTTGTTGTATTTTGGATTTAGATTTTT-3′.

IGF2 Reverse PCR Primer: 5′-[Biotin]-CTCCAAACACCCCCACCTTAA-3′.
IGF2 Sequencing Primer: 5′- GTGGGGAGGGGGTTTATTTTT-3′.
Sequence to be analysed for IGF2:
TTAGGAAGTATAGTTAYGTYGTTTTTTATTGGTTTYGTTA.

- PEG1/MEST (Qiagen):

GeneGlobe Id: PM00126742.
Catalog No.: 978746.
Name: Hs_MEST_04_PM PyroMark CpG assay.
Entrez genes for: ENSG00000106484.
Sequence to be analysed for PEG1/MEST:
AGYGTATGYGTAATYGGTTTTTYGA.

Methylation for each amplicon was calculated as the median of methylation status of each analysed CpG.

### 4.7. Statistical Analysis

Continuous variables are presented as mean ± standard deviation (SD) or median and interquartile range (IQR), as appropriate. Normality was assessed using the Kolmogorov–Smirnov test. Comparisons between two groups were performed using Student’s *t* test or the Mann–Whitney U test, as appropriate. Comparisons among three or more groups were performed using one-way ANOVA or the Kruskal–Wallis test, as appropriate. When applicable, post hoc pairwise comparisons were adjusted using the Bonferroni correction. Categorical variables are presented as counts and/or percentages and compared using Fisher’s exact test. Correlations between variables were assessed using Spearman’s rank correlation coefficient. A two-tailed *p*-value ≤ 0.05 was considered statistically significant.

All computations were carried out with the software R version 4.5.2 (2025 The R Foundation for Statistical Computing, Vienna, Austria. URL https://www.R-project.org/, accessed on 22 November 2025).

## 5. Conclusions

**Figure 2:**
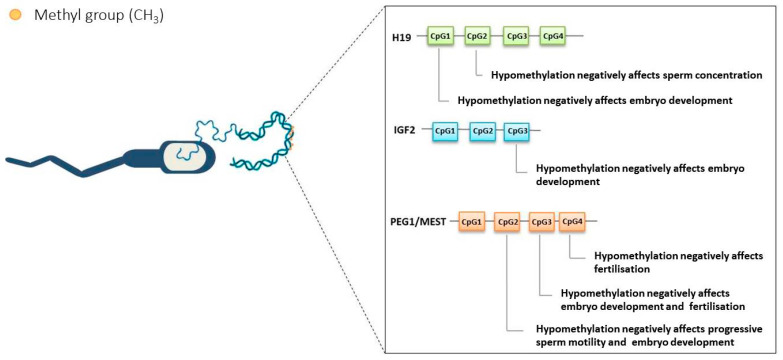
Summary of statistically significant results: methylation of CpG islands H19, IGF2, and PEG1/MEST gene in sperm DNA and their correlations with semen quality and reproductive outcomes.

This pilot study provides preliminary evidence that sperm DNA fragmentation and methylation patterns of key imprinted genes may be associated with ICSI outcomes (Figure 2). A relevant aspect of our study is the evaluation of methylation at specific CpG sites within imprinted gene promoters. This approach allows a more detailed characterisation of epigenetic regulation, as local methylation changes may have functional consequences that are not captured by overall methylation levels. Moreover, by integrating sperm DNA fragmentation with gene-specific methylation analysis in relation to ICSI outcomes, our findings provide a more comprehensive framework for understanding the paternal contribution to early embryonic development. These results suggest that epigenetic alterations and DNA damage may represent interconnected aspects of sperm quality, potentially contributing to impaired reproductive outcomes. Overall, our findings support the growing evidence that molecular and epigenetic signatures of spermatozoa may play a relevant role in ART outcomes. As a pilot study, these results provide preliminary insights and support the need for validation in larger cohorts.

### Strengths and Limitations

Limitations—The main limitation of this pilot study is the relatively small sample size. This reflects the complexity of the study design, which required strict inclusion and exclusion criteria to minimise potential confounding factors, as well as the availability of surplus semen samples from ICSI procedures. Although some correlations reached statistical significance, other findings did not and should be interpreted with caution. These observations should be considered preliminary and require confirmation in larger studies.

Strengths—To date, the literature on the topic remains limited, with most available data derived from animal models or from human studies that do not directly evaluate ART outcomes. To our knowledge, this is one of the few studies examining sperm DNA methylation at the level of individual CpG sites within imprinted genes. Additionally, this study integrates multiple parameters, including sperm DNA fragmentation and clinical parameters such as fertilisation and embryo development in ICSI.

Our findings may contribute to a better understanding of the potential role of spermatozoa in reproductive success, particularly in the context of ICSI.

## Figures and Tables

**Table 1:** Demographic characteristics in three outcome groups; values are expressed as mean ± standard deviation and median (in brackets), with statistical significance in bold.

	No Fertilisation (NF)	Non-Viable Embryo (NVE)	Viable Embryo (VE)	*p*-Value
Male age	47.7 ± 7.2 (46.5)	39.0 ± 5.9 (38.0)	37.7 ± 2.8 ^a^ (37)	**0.039**
Female age	39.8 ± 2.2 (41.0)	36.6 ± 4.1 (37.0)	34.4 ± 3.5 ^a^ (36.0)	**0.020**
Male BMI	29.5 ± 2.2 (29.7)	24.8 ± 1.6 ^a^ (25.8)	26.1 ± 3.2 (26.3)	**0.007**
Female BMI	23.6 ± 3.9 (24.2)	21.5 ± 3.8 (20.7)	23.3 ± 3.1 (22.6)	0.584
Male smoke	0/6	3/7	3/7	0.159
Female smoke	0/6	4/7	1/7	**0.043**

^a^ *p* < 0.05 vs. No Fertilisation (Bonferroni-adjusted).

**Table 2:** Semen parameters, sperm vitality, and sperm DNA fragmentation (SDF) in three groups; values are expressed as mean ± standard deviation and median (in brackets), with statistical significance in bold and trends in italics.

	No Fertilisation (NF)	Non-Viable Embryo (NVE)	Viable Embryo (VE)	*p*-Value
Sperm Concentration (×10^6^/mL)	65.2 ± 62.1 (48.0)	112.6 ± 53.4 (110.0)	49.1 ± 24.7 (54.0)	*0.068*
Progressive Sperm Motility (%)	25.8 ± 24.0 (17.5)	49.3 ± 10.6 (50.0)	42.9 ± 20.2 (50.0)	0.230
Abnormal Forms (%)	92.5 ± 5.1 (93.0)	90.6 ± 4.2 (92.0)	91.7 ± 4.8 (92.0)	0.754
Sperm Vitality (%)	42.2 ± 31.5 (52.0)	68.1 ± 22.9 (80.0)	70.4 ± 13.9 (73.0)	0.124
SDF (%)	29.7 ± 15.6 (33.0)	11.1 ± 5.6 (10.5)	15.3 ± 14.9 (8.4)	**0.014**

**Table 3:** Spearman correlations between semen parameters and sperm DNA fragmentation (SDF) with viable embryos and fertilisation; with statistical significance in bold and trends in italics.

		Viable Embryo	Viable Embryo (adj)	Fertilisation	Fertilisation (adj)
Sperm Concentration (×10^6^/mL)	*Rho*	−0.346	−0.426	0.189	0.176
	*p*-value	0.135	*0.078*	0.424	0.484
Progressive Sperm Motility (%)	*Rho*	0.074	−0.057	0.374	0.499
	*p*-value	0.757	0.822	0.104	**0.035**
Abnormal Forms (%)	*Rho*	0.046	0.062	−0.124	−0.248
	*p*-value	0.848	0.808	0.603	0.322
SDF (%)	*Rho*	−0.184	−0.006	−0.452	−0.622
	*p*-value	0.478	0.984	*0.068*	**0.013**

**Table 4:** Methylation status (%) of the four CpG islands in the H19 promoter across the three groups; values are expressed as mean ± standard deviation and median (in brackets).

H19 Gene	No Fertilisation (NF)	Non-Viable Embryo (NVE)	Viable Embryo (VE)	*p*-Value
Methylation CpG 1 (%)	94.0 ± 2.0 (95.0)	95.0 ± 4.0 (95.0)	98.0 ± 2.0 (98.0)	0.098
Methylation CpG 2 (%)	78.0 ± 6.0 (77.0)	81.0 ± 8.0 (81.0)	75.0 ± 7.0 (78.0)	0.465
Methylation CpG 3 (%)	98.0 ± 3.0 (100.0)	99.0 ± 2.0 (100.0)	99.0 ± 1.0 (100.0)	0.754
Methylation CpG 4 (%)	100 (100.0)	100 (100.0)	100 (100.0)	1.000
Total Methylation (%)	93.0 ± 2.0 (93.0)	93.0 ± 2.0 (93.0)	94.0 ± 3.0 (94.0)	0.915

**Table 5:** Correlation between methylation (%) of the three H19 CpG islands and sperm DNA fragmentation (SDF) with fertilisation and viable embryos; with statistical significance in bold and trends in italics.

H19 Gene	SDF (%)		Fertilisation	Fertilisation (adj)	Viable Embryo	Viable Embryo (adj)
Total Methylation (%)	*Rho*	0.034	0.095	0.383	0.064	−0.001
	*p*-value	0.896	0.692	0.117	0.790	0.996
Methylation CpG 1 (%)	*Rho*	0.124	0.184	0.198	0.530	0.521
	*p*-value	0.635	0.438	0.431	**0.016**	**0.027**
Methylation CpG 2 (%)	*Rho*	0.118	0.000	0.107	−0.419	−0.440
	*p*-value	0.664	1.000	0.682	*0.074*	0.077
Methylation CpG3 (%)	*Rho*	0.051	−0.150	−0.417	0.168	0.069
	*p*-value	0.846	0.527	0.085	0.478	0.785

**Table 6:** Methylation status (%) of the three CpG islands present in the promoter of IGF2 gene in the three groups; values are expressed as mean ± standard deviation and median (in brackets).

IGF2 Gene	No Fertilisation (NF)	Non-Viable Embryo (NVE)	Viable Embryo (VE)	*p*-Value
Methylation CpG 1 (%)	72.0 ± 0.1 (69.0)	71.0 ± 4.0 (69.0)	70.0 ± 3.0 (69.0)	0.854
Methylation CpG 2 (%)	85.0 (86.0)	87.0 (87.0)	87.0 ± 3.0 (89.0)	0.308
Methylation CpG 3 (%)	93.0 (91.0)	94.0 (94.0)	96.0 ± 2.0 (97.0)	0.076
Total Methylation (%)	84.0 ± 0.0 (82.0)	84.0 ± 1.0 (83.0)	85.0 ± 2.0 (85.0)	0.637

**Table 7:** Correlation between methylation (%) of the three IGF2 CpG islands and sperm DNA fragmentation (SDF) with fertilisation events and viable embryos; with statistical significance in bold and trends in italics.

IGF2 GENE	SDF (%)		Fertilisation	Fertilisation (adj)	Viable Embryo	Viable Embryo (adj)
Total Methylation (%)	*Rho*	−0.467	0.095	−0.105	0.164	0.118
	*p*-value	*0.068*	0.690	0.680	0.488	0.640
Methylation CpG 1 (%)	*Rho*	−0.489	0.104	−0.277	0.073	−0.125
	*p*-value	*0.055*	0.661	0.266	0.760	0.621
Methylation CpG 2 (%)	*Rho*	−0.123	0.400	0.308	0.329	0.372
	*p*-value	0.649	*0.081*	0.213	0.157	0.128
Methylation CpG 3 (%)	*Rho*	0.113	0.410	0.281	0.494	0.484
	*p*-value	0.678	*0.073*	0.259	**0.027**	**0.042**

**Table 8:** Methylation status (%) of the four PEG1/MEST CpG islands present in the three groups; values are expressed as mean ± standard deviation and median (in brackets), with statistical significance in bold.

PEG1/MEST Gene	No Fertilisation (NF)	Non-Viable Embryo (NVE)	Viable Embryo (VE)	*p*-Value
Methylation CpG 1 (%)	0.0 (0.0)	1.0 (1.0)	1.0 ± 1.0 (1.0)	0.141
Methylation CpG 2 (%)	2.0 (2.0)	3.0 (2.0)	3.0 ± 1.0 (2.0)	0.123
Methylation CpG 3 (%)	2.0 (2.0)	3.0 (3.0)	3.0 ± 1.0 ^a^ (3.0)	**0.019**
Methylation CpG 4 (%)	1.0 (1.0)	3.0 (3.0)	3.0 ± 1.0 (3.0)	0.659
Total Methylation (%)	1.0 (1.0)	2.0 (2.0)	2.0 ^a^ (2.0)	**0.007**

^a^ *p* < 0.05 vs. No Fertilisation (Bonferroni-adjusted).

**Table 9:** Correlation between the four PEG1/MEST CpG islands and sperm DNA fragmentation (SDF) with fertilisation events and viable embryos; statistical significance is in bold and trends are in italics.

PEG1/MEST Gene		SDF (%)	Fertilisation	Fertilisation (adj)	Viable Embryo	Viable Embryo (adj)
Total Methylation (%)	*Rho*	−0.362	0.644	0.649	0.555	0.503
	*p*-value	0.895	**0.002**	**0.004**	**0.011**	**0.033**
Methylation CpG 1 (%)	*Rho*	0.094	0.347	0.514	0.206	0.176
	*p*-value	0.718	0.133	**0.029**	0.383	0.485
Methylation CpG 2 (%)	*Rho*	−0.461	0.378	0.348	0.496	0.466
	*p*-value	*0.062*	0.101	0.157	**0.026**	*0.051*
Methylation CpG 3 (%)	*Rho*	0.018	0.707	0.752	0.551	0.441
	*p*-value	0.946	**0.000**	**<0.001**	**0.012**	*0.067*
Methylation CpG 4 (%)	*Rho*	−0.367	0.614	0.548	0.364	0.203
	*p*-value	0.147	**0.004**	**0.019**	0.115	0.419

## Data Availability

The original contributions presented in this study are included in the article. Further inquiries can be directed to the corresponding author.

## Abbreviations

The following abbreviations are used in this manuscript:

ART	Assisted Reproductive Technique
BMI	Body Mass Index
CTCF	CCCTC-Binding Factor
DMR	Differentially Methylated Region
ICR	Imprinting Control Region
ICSI	Intracytoplasmic Sperm Injection
IGF2	Insulin-Like Growth Factor 2
NF	No Fertilisation
NVE	Non-Viable Embryo
PEG1/MEST	Paternally Expressed Gene 1/Mesoderm-Specific Transcript
SCD	Sperm Chromatin Dispersion
SCSA	Sperm Chromatin Structure Assay
SDF	Sperm DNA Fragmentation
TUNEL	Terminal Deoxynucleotidyl Transferase dUTP Nick End Labelling
VE	Viable Embryo
